# The complete mitogenome of *Orcula dolium* (Draparnaud, 1801); ultra-deep sequencing from a single long-range PCR using the Ion-Torrent PGM

**DOI:** 10.1186/s41065-017-0028-2

**Published:** 2017-04-04

**Authors:** D. S. J. Groenenberg, J. Harl, E. Duijm, E. Gittenberger

**Affiliations:** 1 Naturalis Biodiversity Center, P.O. Box 9517, 2300 RA Leiden, The Netherlands; 2 Central Research Laboratories, Museum of Natural History Vienna, Vienna, Austria; 3 Department of Biology, Shinshu University, Matsumoto, Japan

**Keywords:** Stylommatophora, Orthurethra, Mitochondrial genome, Gene arrangement

## Abstract

**Background:**

With the increasing capacity of present-day next-generation sequencers the field of mitogenomics is rapidly changing. Enrichment of the mitochondrial fraction, is no longer necessary for obtaining mitogenomic data. Despite the benefits, shotgun sequencing approaches also have disadvantages. They do not guarantee obtaining the *complete* mitogenome, generally require larger amounts of input DNA and coverage is low compared to sequencing with enrichment strategies. If the mitogenome could be amplified in a single amplification, additional time and costs for sample preparation might outweigh these disadvantages.

**Results:**

A sequence of the complete mitochondrial genome of the pupilloid landsnail *Orcula dolium* is presented. The mitogenome was amplified in a single long-range (LR) PCR and sequenced on an Ion Torrent PGM (Life Technologies). The length is 14,063 nt and the average depth of coverage is 1112 X. This is the first published mitogenome for a member of the family Orculidae. It has the typical metazoan makeup of 13 protein coding genes (PCGs), 2 ribosomal RNAs (12S and 16S) and 22 transfer RNAs (tRNAs). *Orcula* is positioned between *Pupilla* and the Vertiginidae as the sister-group of *Gastrocopta* and *Vertigo*, together. An ancestral gene order reconstruction shows that Orthurethra in contrast to other Stylommatophora, have tRNA-H before tRNA-G and that the gene order in the ‘non-achatinoid’ clade is identical to that of closely related non-stylommatophoran taxa.

**Conclusions:**

We show it is feasible to ultra-deep sequence a mitogenome from a single LR-PCR. This approach is particularly relevant to studies that have low concentrations of input DNA. It results in a more efficient use of NGS capacity (only the targeted fraction is sequenced) and is an effective selection against nuclear mitochondrial inserts (NUMTS). In contrast to previous studies based in particular on 28S, our results indicate that phylogeny reconstructions based on complete mitogenomes might be more suitable to resolve deep relationships within Stylommatophora. Ancestral gene order reconstructions reveal rearrangements that characterize systematic groups.

**Electronic supplementary material:**

The online version of this article (doi:10.1186/s41065-017-0028-2) contains supplementary material, which is available to authorized users.

## Background

A recent increase in the number of sequenced mitogenomes allows for a better understanding of gastropod evolution [1–3]. Of the more than sixty mitogenomes that are currently available for Gastropoda, less than twenty belong to the Eupulmonata sensu [4], of which the clade Stylommatophora represents the majority of the terrestrial snails. Although the first stylommatophoran mitogenome was sequenced nearly two decades ago [5], it took more than 15 years before new mitogenomes were added to this group on about a yearly basis, as is the case at present. Complete mitogenomes have been obtained for 18 species of Stylommatophora now (excluding *Euhadra* and *Orcula*; accessed 2016-10-17) (Table 1). These include representatives of the superfamilies Achatinelloidea, Clausilloidea, Helicoidea, Orthalicoidea, Pupilloidea, Succinoidea and Urocoptoidea, or more inclusively the subclades Elasmognatha, Orthurethra, the ‘Limacoid clade’, the informal group Sigmurethra sensu [6] and the ‘achatinoid clade’ sensu [7]. Here we report the mitochondrial genome of a fifth orthurethran species, the first one for the family Orculidae, viz. *Orcula dolium* (Drapernaud, 1801). It is the type species of the genus *Orcula* Held 1837, which comprises 13 species featuring ovate–cylindrical shells of 5 to 10 mm height. Of these, *O. dolium* shows the widest distribution and is ecologically most tolerant. *Orcula* is common in limestone areas of the Central European Alps and the Western Carpathians and is usually associated with mountainous forest habitats and rocky landscapes. Its altitudinal distribution covers a range from 200 m to 2160 m above sea level [8]. Loess sediments of the Pannonian Basin (Hungary, Republic of Croatia and Republic of Serbia) [9–11] and the periphery of the Western and Eastern Alps [12, 13] show that *O. dolium* was also widely distributed throughout glacial periods of the Late Pleistocene.

**Table 1:** List of included mitogenomes (accessed 2016-10-17)

Species	Length (nt)	GenBank	Reference
Stylommatophora			
Orthurethra			
*Achatinella mustelina*	16,323	KU525108	[41]
*Gastrocopta cristata*	14,060	KC185403	[42]
*Orcula dolium*	14,063	KJ867421	This study
*Pupilla muscorum*	14,149	KC185404	[42]
*Vertigo pusilla*	14,078	KC185405	[42]
Sigmurethra			
*Achatina fulica*	15,057	KJ744205	[59]
*Aegista aubryana*	14,238	NC_029419	[60]
*Aegista diversifamilia*	14,039	KR002567	[61]
*Albinaria caerulea* (Deshayes, 1835)	14,130	NC_001761	[5]
*Camaena cicatricosa*	13,843	KM365408	[48]
*Cernuella virgata*	14,147	KR736333	[62]
*Cerion incanum*	15,177	NC_025645	[57]
*Cepaea nemoralis* Linnaeus, 1758	14,100	NC_001816	[63]
*Cornu aspersum* (Müller, 1774)	14,050	NC_021747	[35]
*Cylindrus obtusus* (Draparnaud, 1805)	14,610	NC_017872	[47]
*Dolicheulota formosensis*	14,237	KR338956	[61]
*Euhadra herklotsi*	?	Z71693- Z71701	[64]
*Mastigeulota kiangsinensis*	14,029	KM083123	[65]
*Naesiotus nux*	15,197	KT821554	[24]
Elasmognatha			
*Succinea putris* Linnaeus, 1758	14,092	NC_016190	[3]
Basommatophora			
Hygrophila			
*Biomphalaria glabrata*	13,670	NC_005439	[66]
*Biomphalaria tenagophila*	13,722	NC_010220	[67]
*Galba pervia*	13,768	NC_018536	[68]
*Physella acuta*	14,490	JQ390525	[69]
Archaepulmonata			
Ellobioidea			
*Myosotella myosotis*	14,246	NC_012434	[70]
*Pedipes pedipes*	16,708	NC_016179	[3]
Systellomatophora			
*Rhopalocaulis grandidieri*	14,523	NC_016183	[3]

Despite being incomplete, the mitogenome of *E. herklotsi* was included because of its relevance to the arrangement of genes within the Helicoidea. The mitogenome of *Radix balthica* [71] was excluded because it is poorly annotated (GenBank accession number HQ330989) and of low quality [68, 69]

## Methods

This study was carried out on a specimen of *Orcula dolium dolium* (RMNH 114169) collected in 2009 in SW Berchtesgaden (Bayern, Germany). DNA was extracted with a Qiagen DNA tissue kit. Total yield of DNA extracted was ~13 ug (DNA conc. 66.7 ng/ul with an elution volume of 200 ul). Using universal barcoding primers [14] a partial sequence (655 nt) of *Cytochrome Oxidase subunit I (COI)* was obtained using the procedure described in [15]. This sequence was used to design specimen specific primers (Orcula_529_COI_F 5′-CTAAGACTATTTGTGTGGTCGATCTTA-3′ and Orcula_336_COI_R 5′-TCTAGACCTAATCAAAAGAACAAATGAAG-3′) to amlify the complete mitogenome of *O. dolium*. An amplicon with a length of 13,871 nt was obtained with GoTaq long PCR Master Mix (Promega) using the manufacturers protocol. Thermocycling profile was 2 m. at 94 °C, followed by 40 cycles of 30 s. at 94 °C, 15 m. at 65 °C, 10 m. at 72 °C. The PCR product was gel purified with the Wizard SV gel and PCR cleanup-system (Promega) and subsequently checked on a BioAnalyzer 2100 using a DNA 12000 chip (Agilent). The *Orcula* library was part of a combined run in which different samples were pooled. The purified amplicon was enzymatically digested and individual samples were ligated with a unique Ion Express Barcode Adapter (Life Technologies) using the NEBNext Fast DNA & Library Prep Set for Ion Torrent (New England Biolabs), following the manufacturers instructions. After ligation samples were quantified on the Bioanalyser 2100 using a DNA High sensitivity chip (Agilent). An equimolair pool was prepared of the highest possible concentration. This equimolair pool was diluted according to the calculated template dilution factor to target 10–30% of all positive Ion Sphere Particles. Template preparation and enrichment was carried out with the Ion Touch 2 system, using the OT2 400 kit (Life Technologies), according to the manufactures protocol (7218RevA0). The enriched Ion Sphere Particles were prepared for sequencing on a Personal Genome Machine (PGM) with the Ion PGM 400 Sequencing kit as described in the protocol using a 316v2 chip.

### Quality check and assembly

Reads from the *Orcula* library were separated from the pool based on their unique barcode tag by the Ion Torrent Server. Quality was checked using FastQC (http://www.bioinformatics.babraham.ac.uk/projects/fastqc/). Reads shorter than 35 nt or with a phred score below 28 were removed with Fastx_trimmer (http://hannonlab.cshl.edu/fastx_toolkit/). A ‘de novo’ assembly was carried out with Spades v.2.5.1. [16] and reads were mapped against available orthurethran sequences (KC185403-05, KU525108) using Geneious v.7.1.7 [17]. Finally ‘de novo’ contigs larger than 1 kb (for gene order assessment) and mapping assemblies were merged (see Additional file 1: Figure S1) and used as reference for iterative mapping (medium sensitivity, no fine tuning, not trimmed before mapping; other parameters left at default) of the quality checked reads (again using Geneious v.7.1.7). The reason for this two-step approach was that the initial ‘de novo’ assembly did not result in a contig of expected length. To confirm the final assembly (and enclosed gene order) a selection of matching reads (~ten fold downsampling) was analysed with another assembler, MITObim v. 1.8 [18] (using the 655 nt *COI* sequence as seed bait) and with Spades 2.5.1. [17] again also.

### Annotation

The position of the protein coding genes (PCGs) was determined by alignment with available stylommatophoran mitogenomes (Table 1) and by locating start and stop codons. The identification of the ribosomal RNAs was done with BLAST searches. Arwen and Mitos [19] were used to locate the tRNAs and the secondary structures were all generated with Arwen [20]. The contig sequence was annotated in Geneious [17].

### Phylogenetic analyses

A prerequisite for understanding (mitochondrial) gene rearrangements is a robust, well rooted phylogeny. Representatives of the clades Hygrophila and Eupulmonata were selected as outgroup because these taxa are supposed to be closely related to, but not part of the ingroup [3, 4, 21]. For each of the 13 PCGs alignments were made with TranslatorX [22]; a program that aligns nucleotide sequences based on their corresponding amino acid translations. Translator X was run with the MAFFT alignment module [23] and the invertebrate mitochondrial genetic code; other settings were left at default. A supposed ‘copy’ of ND4L [24] for *Naesiotus nux* (GenBank accession number NC_028553) was excluded because there are no stylommatophoran homologs known to align it with. Ribosomal genes were individually aligned in Geneious, again using MAFFT [23] and conserved datablocks were selected with Gblocks [25] using default settings. The PCG and ribosomal alignments were concatenated in Geneious and exported as a phylip formatted matrix (27 taxa, 13,419 nucleotides). With 41 datablocks specified (each codon position and the ribosomal RNAs), PartitionFinder v.1.1.1 [26] estimated the best partitioning scheme (33 partitions) and nucleotide substitution models. A Bayesian analysis (two simultaneous MCMC runs 10 M generations each) was conducted with MrBayes 3.2.3 hosted on the CIPRES Science Gateway [27]. Inspection of the parameter files with Tracer v.1.5 [28] showed proper mixing of the MCMC (effective sampling size values > 200). The first 2500 trees (25% of each tree-file) were discarded as burnin. Majority rule consensus trees were visualised and edited in FigTree v.1.4.0 [29]. The procedure was repeated with the amino acid alignments (13 datablocks, 4190 amino acids) from TranslatorX [22]. The estimated partitioning scheme now consisted of six partitions and the analysis was carried out with 2.5 M generations to keep computation time tenable. Nucleotide and amino acid datamatrices were analysed separately to see if both would yield phylogenies with similar topologies. Abbreviations: BPP = Bayesian Posterior Probability.

### Mitochondrial gene arrangements

An ancestral gene order reconstruction [30] was done using the Maximum Likelihood Gene Order analysis (MLGO) web server (http://www.geneorder.org/server.php) using the phylogeny obtained in the previous step (outgroup reduced to *Biomphalaria*) as a fixed tree (ie. Small Parsimony Problem, using SPP option).

### Taxonomic implications

For the classification and the nomenclature of the various taxa, the review by Bouchet and Rocroi [6] served as the primary starting point. Historic classifications such as the Pilsbry-Baker system [31, 32] are not exhaustively discussed. The more recent literature is dealt with only when it contains additional data. Ever since the rise of phylogenetic systematics, the increase of more detailed anatomical analyses and in particular the quickly growing quantity of molecular data, the classification of the gastropods has been in a confusing transitional state. In the monograph of Zilch [33] for example, published before the rise of phylogenetic systematics had started, the classis Gastropoda is subdivided into the subclasses Prosobranchia and Euthyneura. The latter subclassis contains the ordines Basommatophora and Stylommatophora, whereas the ordo Stylommatophora is further split into the subordines Orthurethra, Heterurethra and Sigmurethra. Many of these names are still in use, but often not for exactly the same group of species. Next to the classical taxonomical categories, new nominal taxa names were more recently introduced, like ‘clade’ and ‘informal group’. Bouchet et al. [6] for example, in a recent nomenclatorial monograph on gastropod classification and nomenclature, use ‘Informal Group Pulmonata’ as a partial synonym of Zilch’s Euthyneura. The Orthurethra are accepted by [6] as a ‘Subclade’, which is identical with Zilch’s subordo of that name. There are many more discrepancies, however. For a more recent contribution to this subject, see [4]. Here we do not aim at a summary of the changing and sometimes conflicting views regarding gastropod phylogeny.

## Results

**Fig. 1:**
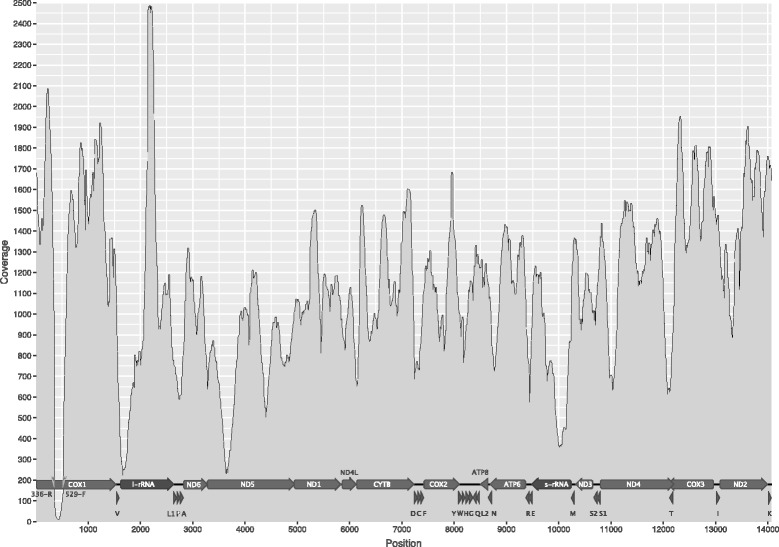
Coverage plot of the *Orcula dolium* mitochondrial genome (14,063 nt) and corresponding gene annotation

The Ion-Torrent run resulted in 118,854 reads of which 115,412 were retained after trimming with Fastx_trimmer. The retained reads were in the length range 35–313 nt and had an average length of 143.8 nt (sd. 42.8). Of these 107,569 could be mapped against the merged reference sequence, resulting in an average coverage of 1112X (min. 208X, max. 2483X; see Fig. 1). The mitogenome of *O. dolium* has a length of 14,063 nucleotides, which is within the known range for Stylommatophora (13.843 and 16.323 nt for *Camaena* and *Achatinella*, respectively) and very similar to that reported for other Pupilloidea (14,060 and 14,078 nt for *Gastrocopta* and *Vertigo* respectively). Both MITObim v.1.8 [18] and Spades v.2.5.1 [17] yielded the same length and nucleotide sequence using the downsampled dataset. The AT content is 66% and skews for AT and GC are −0.084 and 0.043. All 37 common metazoan genes (13 protein coding genes, two rRNAs and 22 tRNAs) were recovered. The gene order of PCGs and ribosomal RNAs was identical to those of most Stylommatophora (Achatinelloidea and certain Helicoidea excepted). The distribution of tRNAs was, with one mutational step, most similar to that of *Vertigo* and *Albinaria*. All tRNAs showed standard cloverleaf secondary structures except for tRNA-K and tRNA-W which had missing T-arms and tRNA-S1 which had a D-arm missing (see Additional file 2: Figure S2). The annotated mitogenome of *O. dolium* has been deposited in GenBank (accession number KJ867421).

## Discussion

### Selection of outgroup

Recent studies have shown that the traditionally accepted nominal taxa Pulmonata and Opisthobranchia are not monophyletic, what necessitated a new classification of the Euthyneura [4]. Within that taxon these authors accept Panpulmonata and Eupulmonata, with the Stylommatophora belonging to the Eupulmonata. The Informal Group Basommatophora is assigned to a paraphyletic group Panpulmonata. Next to the selection of markers and taxa, outgroup selection strongly affects our concept of Euthyneuran phylogeny, as is evident from the literature [1, 3, 4, 21, 34–36]. Despite the differences, all these studies show that the clades Hygrophila (Lymnaeoidea & Planorboidea), Systellommatophora (Veronicelloidea) and Eupulmonata (Ellobioidea) are closely related to, but separate from the Stylommatophora (i.e. these are eligible outgroup taxa).

### Gene order

**Fig. 2:**
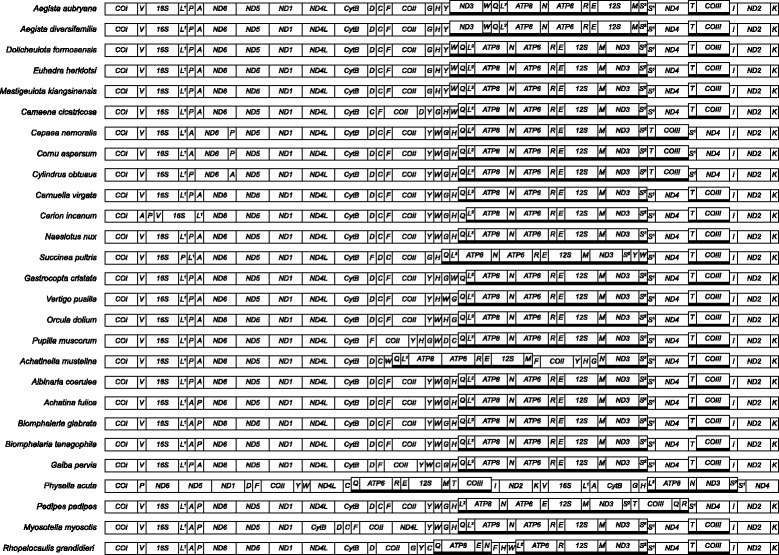
Mitogenomic gene arrangement of Stylommatophora and Euthyneuran outgroup taxa

Changes in mitochondrial gene order are common in gastropods [1, 3, 21]. Among the Stylommatophora however, the order is rather conserved (Fig. 2). Here most shifts occur in the arrangement of tRNAs. In the Stylommatophora PCG rearrangements have only been recorded in Helicidae (*COIII)*, *Aegista* (Bradybaenidae; *ND3*) and *Achatinella* (Achatinellidae; *COII*). Most transpositions have been observed in the Helicoidea, but there is a strong bias in available data for that group.

### Polarisation

**Fig. 3:**
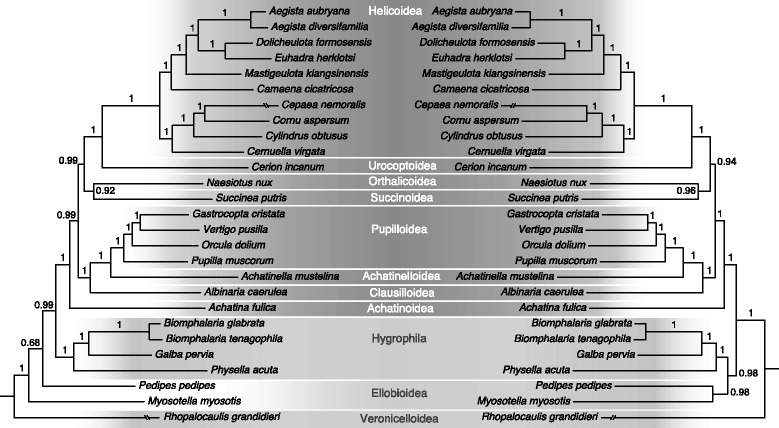
Bayesian mitogenomic phylogeny reconstructions of Stylommatophora based on nucleotide (*left*) and amino acid data (*right*)

**Fig. 4:**
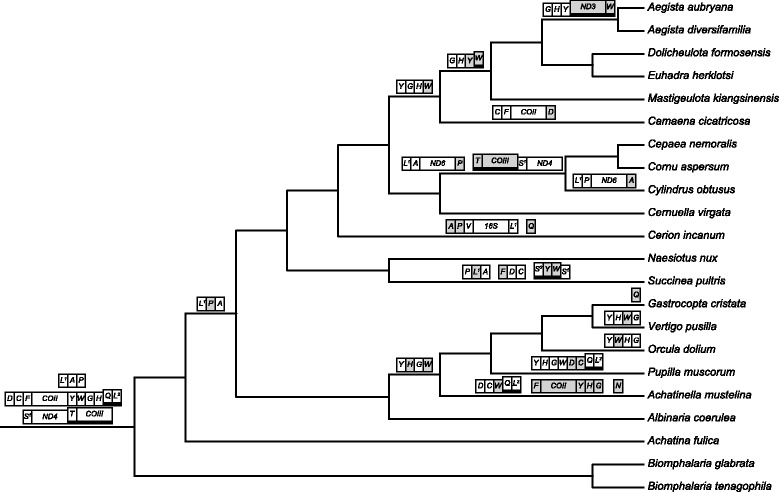
Ancestral (mt) gene order reconstruction for Stylommatophora

The monophyly of the Stylommatophora has been shown repeatedly [4, 7, 34, 37] (Fig. 2), but a subdivision into lower taxa remained problematic. Inclusion of additional genes or investigation of rare genomic changes (RGCs), such as changes in mitochondrial gene order [38] has been suggested [39] to solve this problem. Mitochondrial gene order data (Fig. 2) together with the phylogeny (Fig. 3) allowed for a reconstruction of the ancestral gene order (Fig. 4). Rearrangements that characterize established systematic groups are discussed below.

### Comparison with existing classifications

The number of Stylommatophora with known mitogenomes is still limited, but representatives of most of the major groups are available now. This allows for a preliminary comparison.

#### Orthurethra [31]

Initially, Orthurethra was considered a ‘primitive’ group of Stylommatophora [31, 40]. Molecular analyses showed however, that the Orthurethra are a more derived taxon [7, 37]. Bouchet and Rocroi [6] accept five orthurethran superfamilies, one of which is the Pupilloidea. Complete mitogenomic sequences are available only for Achatinelloidea [41] and for Pupilloidea, i.e. for Pupillidae and Vertiginidae [42] and now additionally for Orculidae.

The Orthurethra and the Pupilloidea are both monophyletic. The Vertiginidae, represented by *Gastrocopta* and *Vertigo* are the sister-group of the Orculidae (Fig. 2). Recently, *Gastrocopta* (Gastrocoptinae) was excluded from the ‘vertiginid’-clade (Vertiginidae), mainly based on 28S sequence data; that phylogeny [43] showed two clades: one consisting of *Pupilla* and *Vertigo* and another with *Orcula* and *Gastrocopta*, albeit poorly supported. These sister-group relationships, as well as the exclusion of Gastrocoptinae from Vertiginidae are rejected by our data (Fig. 2).

The Clausilioidea, classified in an Informal Group Sigmurethra [6] and the Orthurethra are sister-groups (BPP = 1.0; Fig. 2).

In the Orthurethra (and Stylommatophora in general) most mitochondrial gene rearrangements occurred in the region between *CytB* and *ATP8*. Except for that region, the gene order of Pupilloidea is identical to that in *Albinaria* (Fig. 2). In the Achatinelloidea a number of rearrangements was found that are not observed in Pupilloidea (most prominent is the transposition of the region tRNA-F_*COII_*tRNA-YHG; Fig. 2). Both orthurethran superfamilies have the gene order tRNA-HG (or at least tRNA-H before tRNA-G) in which they deviate from other Stylommatophora (which have tRNA-GH). We hypothesize that the arrangement tRNA-HG is an apomorphic character state for the Orthurethra.

The MLGO result predicted tRNA-YHWG as the ancestral gene arrangement for Orthurethra (requiring additional steps for all orthurethrans except *Vertigo*). Alternatively tRNA-YHGW would have been equally parsimonious (also nine steps) and is more likely, given that tRNA-YHG is observed in *Achatinella, Pupilla* and *Gastrocopta*. Therefore the latter scenario was adopted in Fig. 4. In either reconstruction the shift from tRNA-GH to tRNA-H_before_G must have taken place early in orthurethran history. Additionaly, for the Pupilloidea*,* tRNA-W was transposed independently in *Orcula* and *Vertigo,* tRNA-DC in *Pupilla,* and tRNA-Q from L-strand to H-strand in *Gastrocopta* (Figs. 2 and 4)*.*

The mitogenome of *Achatinella mustelina* is said to be “similar to those of other pulmonates” [41]. We assume that statement refers to gene composition, not gene order, because one of the largest transpositions recorded for Stylommatophora is seen in *Achatinella*. The latter taxon has transposed tRNA-F_*COII*_tRNA-YHG (assuming it was not the larger fragement tRNA-WQL^2^_ATP8-ATP6_tRNA-RE_12S), tRNA-W and tRNA-N (Figs. 2 and 4).

#### Heterurethra [31]

The Heterurethra sensu Pilsbry [31] or the subclade Elasmognatha according to [6] when Athoracophoroidea are included next to Succineoidea, were classified in a clade with Acavoidea (*Leucotaenius*) and Orthalicoidea (*Placostylus*) [7, 37], albeit poorly supported. Our analyses support (BPP = 0.92/0.97; Fig. 3) a sister-group relation between Elasmognatha (*Succinea*: Succineoidea) and Orthalicoidea (*Naesiotus*). In agreement with previous studies [7, 37] our data indicate that Succineoidea and Orthalicoidea together are the sister-group of the combined Urocoptoidea and Helicoidea (Fig. 3). Of the three gene rearrangements within *Succinea* (Figs. 2 and 4) none is shared with *Naesiotus*.

A peculiar feature of *Naesiotus nux* (genbank access.nr. KT821554) is a ‘duplication’ of *ND4L* (positioned between tRNA-L and tRNA-P; [24]). Since this ‘copy’ (sequence divergence > 60%) was not present in other Stylommatophora it could not be included in our analyses. It might partly explain the increased size of the mitogenome of *N. nux* compared to that of other Stylommatophora (Table 1).

#### Mesurethra [32]

Of this nominal taxon the Cerionidae and Clausiliidae, which were included by [44], are represented. Earlier studies showed the polyphyly of this group [7, 37]. Our phylogeny reconstructions (Fig. 3) also reject the hypothesis of a close relation between Cerionidae and Clausiliidae.

Recently, Urocoptidae and Cerionidae were shown to constitute a monophyletic group (based on 28S sequence data) for which the superfamily Urocoptoidea was introduced [45]. Urocoptoidea and Helicoidea (= Sigmurethra) are sister-groups (BPP = 1.0; Fig. 3). Vaught [46] classified only the Clausiloidea as Mesurethra.

#### Sigmurethra [31]

The Sigmurethra sensu Pilsbry [31] was already shown to be paraphyletic [7, 37]. Our data reconfirm that Sigmurethra is not monophyletic (Fig. 3). Bouchet and Rocroi [6] refer to the this nominal taxon as an ‘Informal Group’, which may remain in use as long as no preferential alternative has been advocated.

#### ‘Achatinoid clade’ [7, 37]

In our analyses, *Achatina fulica* is the sister-group of the ‘non-Achatinoid’ clade. Stylommatophora (‘Achatinoid’ and ‘non-Achatinoid’ clades) is a monophyletic group (BPP = 1.0; Fig. 3). The mitogenomic gene arrangement of *Achatina* differs from that of the other Stylommatophora (Fig. 2), but it is identical to that of the outgroup genera *Biomphalaria* and *Planorbarius*, which are classified with the Planorboidea of the clade Hygrophila. Unlike the representatives of the ‘non-Achatinoid clade’, *Achatina* has the gene order tRNA-LAP (between *16S* and *ND6*), which also occurs in more distantly related outgroups (*Pedipes, Myosotella* and *Rhopalocaulis*; Fig. 2). Therefore, using the principle of outgroup comparison, we hypothesize that the ancestral mitochondrial gene order of the Stylommatophora has been identical to the arrangement currently found in *Achatina* and in some taxa of the clade Hygrophila.

#### ‘Non-Achatinoid clade’ [7, 37]

Our data show a strongly supported (BPP = 1.0; Fig. 3) basal split in the ‘non-Achatinoid clade’ with on the one hand Orthurethra (Achatinelloidea, Pupilloidea) + Clausilioidea and on the other hand Elasmognatha (= Heterurethra) + Orthalicoidea + Urocoptoidea + Helicoidea. In the latter clade Elasmognatha + Orthalicoidea is the sister-group of Urocoptoidea + Helicoidea.

Rearrangements of PCGs are thus far only observed in the Helicoidea and Achatinelloidea. Separation of *ND6* and *ND5* by a tRNA from the L-P-A region and transposition of tRNA-T + *COIII* might indeed be an apomorphy for the Helicidae, as suggested by [47]; the recently added mitogenome of *Cornu aspersum* [35] confirms this. We hypothesize that the gene arrangement tRNA-GH before tRNA-YW is an apomorphic character state characterizing Bradybaenidae. The transposition of *ND3* is not observed in the other bradybaenids and at the moment being, is unique to *Aegista*. The mitogenomic gene arrangement of Camaenidae might be in between that of an helicoid ancestor and that of Bradybaenidae; tRNA-Y is still at the ancestral position, whereas tRNA-W is already transposed to the ‘bradybaenid’ location (albeit still on the heavy strand). Wang et al. concluded that the mitogenomic gene order of *Camaena cicatricosa* differs from that in other stylommatophores in the position of *COII* and the tRNA’s C, F, D, G, H and W [48]. The single transposition of tRNA-D is a more parsimonious explanation for the first four ‘differences’. The latter three could subsequently be explained by a single transposition of tRNA-W after tRNA-GH or the transposition of tRNA-GH (as a single unit) before tRNA-W. Movement of two consecutive tRNA’s as a single unit is not uncommon, as is exemplified by tRNA-YW in *Succinea* and tRNA-AP in *Cerion* (Fig. 2).

Camaenidae and Bradybaenidae have been considered confamilial by Scott [49], whereas both nominal taxa are paraphyletic according to others [7, 50]. According to our analyses, Camaenidae and Bradybaenidae form a clade within the Helicoidea (Fig. 3), leaving no place for a separate superfamily Camaenoidea, which was accepted by [51, 52].

The mitochondrial gene arrangements of *Albinaria* (Clausilioidea) and *Naesiotus* (Orthalicoidea) are identical, despite the fact that both taxa belong to different basal groups within the ‘non-Achatinoid’ clade. We hypothesize that this is the ancestral mitogenomic gene order of the ‘non-Achatinoid clade’ and that a rearrangement from tRNA-LAP to tRNA-LPA characterized the most recent common ancestor of this clade.

### On the chosen sequence strategy

Amplification of the complete mitochondrial genome in a single LR-PCR with target specific primers for the snail species tested here was straightforward. Nevertheless, other studies show that full mitogenomic amplifications can be stochastic [53] and the method will not work with degraded material. Alternatively the mitochondrial fraction can be enriched by physical isolation (e.g. centrifugation in CsCl-gradient; requires significant amounts of starting material) or by target capture approaches, in which mitochondrial sequences are isolated by hybridisation to biotinylated probes [54]. Some studies abandon mitochondrial enrichment steps entirely and shotgun sequence (pools of) DNA extracts instead [41, 47, 55–58]. Each of these methods have their own strengths and weaknesses. In studies where multiple extracts are pooled and that do not make use of indexing tags, additional controls are necessary to check against ‘chimeric’ assemblies (especially when closely related taxa are involved). To this end (and for linking the obtained mitogenomes to species or vouchers) multiple ‘bait’ sequences have to be determined a priori [55, 56, 58]. A mitogenome assembled with pooled DNA from a large number of individuals of the same species [41], is inevitably artificial. Enrichment of the mitochondrial fraction by LR-PCR (and labeling using indexing tag sequences) is expensive and potentially time-consuming. With the capacity of current generation sequencers mitochondrial enrichment strategies might seem outdated. Advantages of the here demonstrated method are that it ensures obtainment of the *complete* mitochondrial locus from even minute amounts of starting material, there are no negative costs associated with increasing genome sizes (no mitochondrial to nuclear ratio effect) and it diminishes the risk of erroneoulsy sequencing NUMTs.

## Conclusion

This study shows that a complete mitogenome can be amplified and sequenced with high coverage from a single LR-PCR. The approach might be especially relevant in situations where only small amounts of starting material are available. Phylogeny reconstructions based on entire mitogenomes are promising to resolve deep level relationships within Stylommatophora, that could not be resolved using only *28S* sequence data. Well supported groups from previous studies based on ten-fold less sequence data [7, 37] were reconfirmed. The region between *COII* and *ATP8* is apparently a hot spot for rearrangements in stylommatophoran mitogenomes. The ‘Achatinoid’ clade is a basal branch in Stylommatophora and has the same mitochondrial gene arrangement as closely related non-Stylommatophoran taxa (especially from the clade Hygrophila). Rearrangements in mitochondrial gene order can characterize different stylommatophoran ranks, e.g. (non-) ‘Achatinoid’ clade, Orthurethra, Helicidae and Bradybaenidae.

## Additional files

Additional file 1: Figure S1. Assembly strategy. (A) Regions where reads could not be mapped to the reference sequence (B) Regions that broke up the ‘de novo’ assembly (C) Mapping and de novo contigs merged. (PDF 15 kb)

Additional file 2: Figure S2. Secondary structures of the inferred tRNAs of *Orcula dolium*. (PDF 107 kb)
