# Supplementary material for: The complete mitogenome of Orcula dolium (Draparnaud, 1801); ultra-deep sequencing from a single long-range PCR using the Ion-Torrent PGM
Source: Hereditas. 2017 Apr 4;154:7. doi: 10.1186/s41065-017-0028-2 (PMC5379511; doi:10.1186/s41065-017-0028-2)
Supplement: Supplementary file 1 — Assembly strategy. (A) Regions where reads could not be mapped to the reference sequence (B) Regions that broke up the ‘de novo’ assembly (C) Mapping and de novo contigs merged. (PDF 15 kb) [file 41065_2017_28_MOESM1_ESM.pdf]

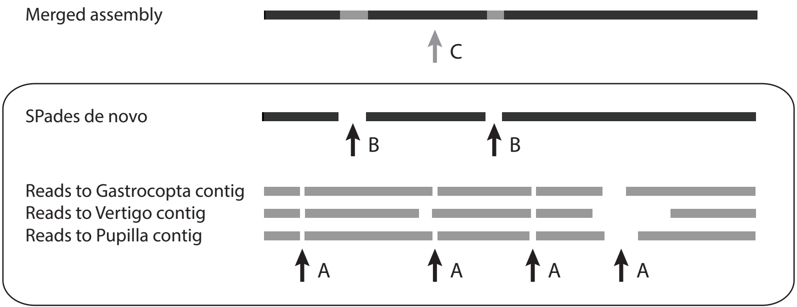
Merged assembly
C
SPades de novo
B
B
Reads to Gastrocopta contig
Reads to Vertigo contig
Reads to Pupilla contig
A
A
A
A
