# Supplementary material for: The complete mitogenome of Orcula dolium (Draparnaud, 1801); ultra-deep sequencing from a single long-range PCR using the Ion-Torrent PGM
Source: Hereditas. 2017 Apr 4;154:7. doi: 10.1186/s41065-017-0028-2 (PMC5379511; doi:10.1186/s41065-017-0028-2)
Supplement: Supplementary file 2 — Secondary structures of the inferred tRNAs of Orcula dolium. (PDF 107 kb) [file 41065_2017_28_MOESM2_ESM.pdf]

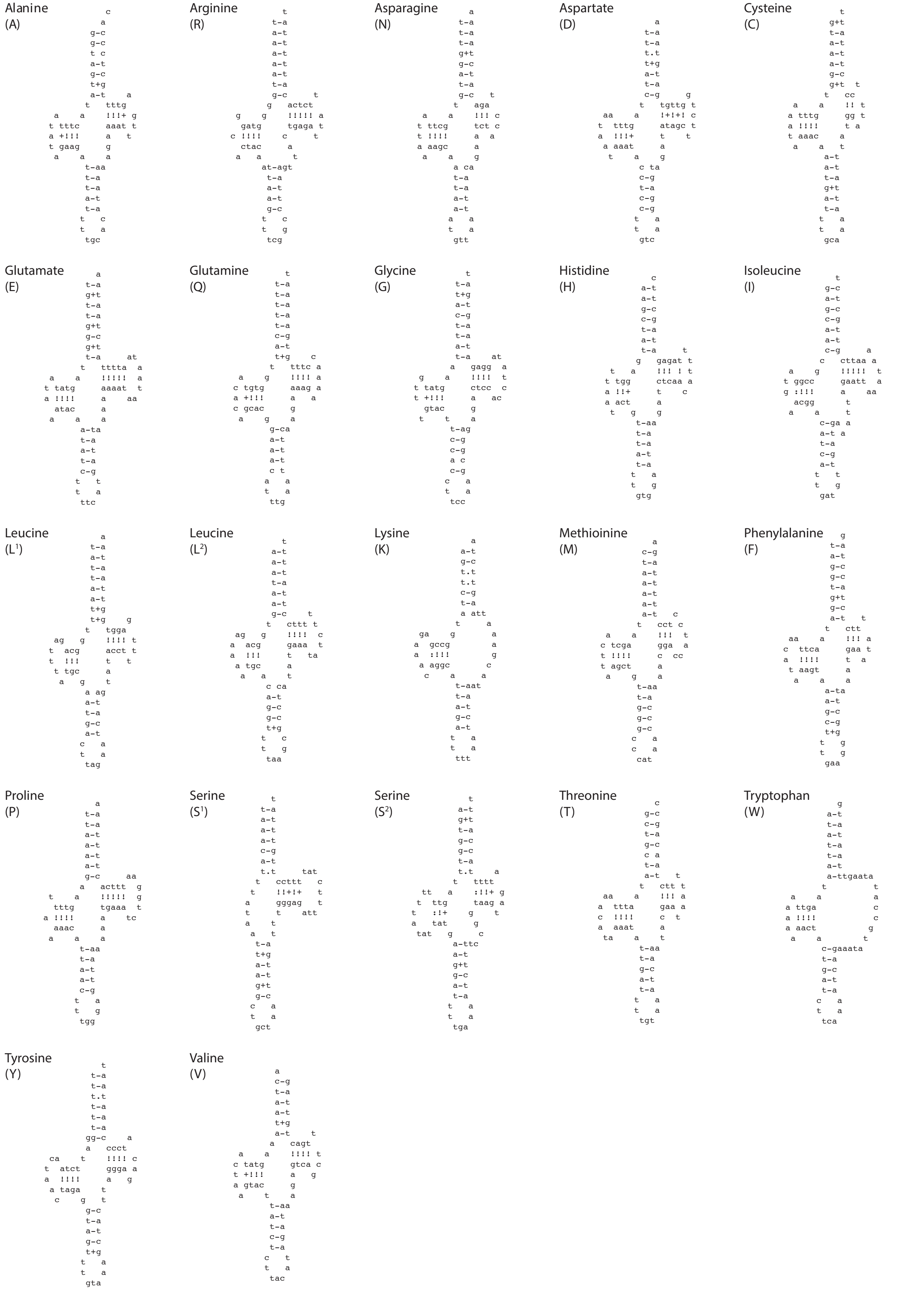

Alanine (A)
Arginine (R)
Asparagine (N)
Aspartate (D)
Cysteine (C)
Glutamate (E)
Glutamine (Q)
Glycine (G)
Histidine (H)
Isoleucine (I)
Leucine (L1)
Leucine (L2)
Lysine (K)
Methioinine (M)
Phenylalanine (F)
Proline (P)
Serine (S1)
Serine (S2)
Threonine (T)
Tryptophan (W)
Tyrosine (Y)
Valine (V)
